# Modeling Latent Topics in Social Media using Dynamic Exploratory Graph Analysis: The Case of the Right-wing and Left-wing Trolls in the 2016 US Elections

**DOI:** 10.1007/s11336-021-09820-y

**Published:** 2021-11-10

**Authors:** Hudson Golino, Alexander P. Christensen, Robert Moulder, Seohyun Kim, Steven M. Boker

**Affiliations:** 1 University of Virginia, Charlottesville, USA; 2 University of Pennsylvania, Philadelphia, USA

**Keywords:** text mining, latent topic analysis, network models, dynamics, time embedding

## Abstract

The past few years were marked by increased online offensive strategies perpetrated by state and non-state actors to promote their political agenda, sow discord, and question the legitimacy of democratic institutions in the US and Western Europe. In 2016, the US congress identified a list of Russian state-sponsored Twitter accounts that were used to try to divide voters on a wide range of issues. Previous research used latent Dirichlet allocation (LDA) to estimate latent topics in data extracted from these accounts. However, LDA has characteristics that may limit the effectiveness of its use on data from social media: The number of latent topics must be specified by the user, interpretability of the topics can be difficult to achieve, and it does not model short-term temporal dynamics. In the current paper, we propose a new method to estimate latent topics in texts from social media termed *Dynamic Exploratory Graph Analysis* (DynEGA). In a Monte Carlo simulation, we compared the ability of DynEGA and LDA to estimate the number of simulated latent topics. The results show that DynEGA is substantially more accurate than several different LDA algorithms when estimating the number of simulated topics. In an applied example, we performed DynEGA on a large dataset with Twitter posts from state-sponsored right- and left-wing trolls during the 2016 US presidential election. DynEGA revealed topics that were pertinent to several consequential events in the election cycle, demonstrating the coordinated effort of trolls capitalizing on current events in the USA. This example demonstrates the potential power of our approach for revealing temporally relevant information from qualitative text data.

## Introduction

The past few years were marked by increased online offensive strategies perpetrated by state and non-state actors to sow discord and promote the questioning of the legitimacy of democratic institutions in the US and Western Europe (Taddeo, 2017; Ziegler, 2018). These offensive strategies ranged from traditional cyber-attacks (e.g., denial-of-service, data leaking, and application compromising; Hernandez-Suarez et al., 2018) to information warfare—a set of tactics and operations involving the protection, manipulation, degradation, and denial of information (Libicki, 1995). The goals of information warfare are to attack an adversary’s knowledge and belief systems (Szafranski, 1995), fabricate false or distorted stories, generate opposition movements, and destabilize an adversary (Ziegler, 2018). In the past decade, the increase in the number of people using social media platforms (such as Twitter) and sharing content online (over 1 billion posts per month around the world; Hernandez-Suarez et al., 2018) has led to increased gains from information operations on both scale and impact.

A recent notable information operation was to convert online activities in social media to influence the public opinion and voters in the USA during the 2016 campaign, with attacks occurring before and during the electoral process (Linvill & Warren, 2018). Social media accounts linked to the Internet Research Agency (IRA), based in Russia, were used to sow discord into the US political system, using trolls and robots that masqueraded as American citizens to try to divide voters on a wide range of issues (Linvill & Warren, 2018). Qualitative analysis of the content published by IRA-linked Twitter accounts has been conducted elsewhere (see Linvill et al., 2019), yet relatively little insights have been gained despite the large amount of information posted by the trolls (almost 3 million tweets from 2848 Twitter handles).

Quantitative analysis of texts, however, can be very useful in understanding the content of the trolls’ online posts and for uncovering some of their communicative strategies. Among the quantitative techniques used, topic modeling is one of the most widely applied (e.g., Ghanem et al., 2019; Zannettou et al., 2019a; Zannettou et al., 2019b), generally implemented using *latent Dirichlet allocation* (LDA; Blei et al., 2003). Despite being one of the most well-known and widely used topic modeling techniques, LDA has some limitations: (1) the number of latent estimated topics must be specified by the user, (2) interpretability can be difficult to achieve (e.g., probabilities for each topic must be interpreted), (3) it assumes that the topics are uncorrelated, and (4) it does not model short-term temporal dynamics.

In the current paper, we propose a new method to estimate latent dimensions (e.g., topics) in multivariate time series termed *Dynamic Exploratory Graph Analysis* (DynEGA). The DynEGA technique can be used to estimate the latent structure of topics published in social media (using time series of word frequencies), improving our capacity to understand and summarize the content posted by accounts created as tools of information warfare. Ultimately, quantitative modeling of text content can help uncover some communicative strategies used in online information operations. The DynEGA approach uses time delay embedding to pre-process each variable (e.g., time series of words counts) and estimates the derivatives from each variable using *generalized local linear approximation* (GLLA; Boker et al., 2010). Finally, a network psychometrics approach for dimensionality assessment termed *exploratory graph analysis* (EGA; Golino & Epskamp, 2017; Golino et al., 2020b) is used to identify clusters of variables that are changing together (i.e., dynamic latent topics). The DynEGA approach automatically estimates the number of latent topics and their short-term temporal dynamics with the results displayed as a network plot, which facilitates their interpretation. Importantly, the DynEGA approach does not assume that the topics are uncorrelated (e.g., LDA and other commonly applied topic modeling techniques), it can accommodate different time scales, and it can estimate the latent structure at different levels of analysis (i.e., population, groups, and individuals).

After describing the DynEGA method, we compare it to LDA in a brief simulation study where the latent topics are simulated using the direct autoregressive factor score model (**DAFS**; Engle & Watson, 1981; Nesselroade et al., 2002), which is characterized by the autoregressive structure of latent dimensions (Nesselroade et al., 2002). In the simulation, LDA is implemented using different estimation methods via the topicmodels package (Hornik & Grün, 2011), and the number of latent topics is verified using AIC, BIC, and the algorithms developed by Arun et al. (2010), Cao et al. (2009), and Deveaud et al. (2014). We also investigate the impact of the number of embedded dimensions used in GLLA on the estimation of the number of simulated topics and propose an approach to tune this hyperparameter using the total entropy fit index (Golino et al., 2020a).

Finally, we apply the DynEGA method to the Twitter data published by Linvill and Warren (2018), which contains posts from IRA-linked accounts that were identified as right- and left-wing trolls. The goals of the current paper are to introduce the new dynamic EGA model, verify its suitability to estimate latent topics in a brief simulation study, and investigate the strategies used by right- and left-wing trolls to sow discord in the US political system. Being able to identify the communication strategies used by the IRA can potentially enhance our capacity to understand online intelligence operations, which are likely part of information warfare efforts perpetrated by both state and non-state actors. We have implemented the DynEGA method into the *EGAnet* package for the R software environment (Golino & Christensen, 2019; Golino et al., 2020b; R Core Team, 2018). All codes used in the current paper are available on the Open Science Framework platform for reproducibility purposes: https://osf.io/4ya6x/?view_only=b6078b404e3049818b359ae0d514f966.

## Exploratory Graph Analysis: A (Very) Brief Overview

The origins of network models in psychology can be traced back to the seminal work of Cattell in the mid-60’s (Boker, 2018; Cattell, 1965) and less explicitly to the proposition of image structural analysis by Guttman (1953). It gained more traction, however, after the publication of the mutualism model of intelligence (Van Der Maas et al., 2006) and the proposition of the network perspective of psychopathological constructs (Borsboom, 2008; Borsboom & Cramer, 2013; Cramer et al., 2010; Fried et al., 2017). Network models have since been applied in clinical (Bork et al., 2018), cognitive (Golino & Demetriou, 2017; Van Der Maas et al. 2017), social (Dalege et al., 2017), and many other areas of psychology (Epskamp et al., 2017).

The rapid developments of network modeling in psychology spawned a new subfield of quantitative psychology termed *network psychometrics* (Epskamp, 2018). In these models, nodes (circles) represent variables and edges (lines) represent associations between the nodes. Under this framework, Golino and Epskamp (2017) proposed the use of network psychometrics as a method for dimensionality assessment and termed this novel approach exploratory graph analysis. Unlike previous factor analytic methods, EGA produces a visual guide—network plot—that not only indicates the number of dimensions to retain, but also which nodes (e.g., items) cluster together and their level of association. Simulation studies have shown that EGA presents comparable or better accuracy than the state-of-the-art parallel analysis technique when estimating the number of simulated factors (Christensen, 2020; Golino & Epskamp, 2017; Golino et al., 2020b).

The EGA approach currently uses two network estimation methods (for a review, see Golino et al., 2020b): graphical least absolute shrinkage and selection operator (*GLASSO*; Friedman et al., 2008) and triangulated maximally filtered graph (TMFG; Massara et al., 2016). After the network is estimated, an algorithm for community detection on weighted networks is used (Walktrap; Pons & Latapy, 2005). The next sections will briefly introduce the network estimation methods and the community detection algorithm used in EGA.

### Graphical LASSO

The GLASSO (Friedman et al., 2008) is the most commonly applied network estimation method in the psychometric network literature. Networks estimated using the GLASSO method are a Gaussian graphical model (GGM; Lauritzen, 1996), where edges represent partial correlations between variables after conditioning on all other variables in the network. The least absolute shrinkage and selection operator (*LASSO*; Tibshirani, 1996) is used to control for spurious relationships and shrink coefficients to zero, generating a sparse and more parsimonious network. The GLASSO procedure can be tuned by generating multiple networks, with different levels of regularization (i.e., from a fully connected network to a fully unconnected network). This approach is termed the *GLASSO path*, in which GLASSO is run for *n* values using the tuning parameter $\lambda$. For each estimated network, the extended Bayesian information criterion (EBIC; Chen & Chen, 2008) is computed and the graph with the best EBIC is selected (Epskamp et al., 2018a; Epskamp & Fried, 2018; Foygel & Drton, 2010). The EBIC has a hyperparameter ($\gamma$) that controls the severity of the model selection (i.e., how much the EBIC prefers simpler models; Epskamp & Fried, 2018). Most commonly, $\gamma$ is set to 0.5 (Foygel & Drton, 2010), although greater sensitivity (true-positive proportion of edges) can be gained from lower $\gamma$ values (e.g., $\gamma = 0$) at the cost of specificity (true-negative proportion of edges; Williams & Rast, 2019). In EGA, the network estimation starts with $\gamma = 0.5$, but if the resulting network has disconnected nodes, then $\gamma$ is set to 0.25 and then to 0 if nodes are still disconnected. When $\gamma$ is zero, EBIC equals the Bayesian information criterion (Foygel & Drton, 2010).

### Triangulated Maximally Filtered Graph

The triangulated maximally filtered graph (TMFG; Massara et al., 2016) is another network estimation method that has been used in the psychometric network literature (e.g., Christensen, Kenett, Aste, Silvia, & Kwapil, 2018; Golino et al., 2020b). The TMFG algorithm starts by identifying the four variables that have the highest sum of correlations to all other variables in the zero-order correlation matrix. Using their respective correlation, the four variables are connected to one another (with their zero-order correlations as weights) forming a tetrahedron. This tetrahedron is the beginning of the network. Next, the algorithm identifies a variable that is not included in the tetrahedron and adds the variable that maximizes its sum of correlations to three of the variables already in the network. This variable is then connected to the those variables (with their zero-order correlations as weights). This process continues iteratively until every variable is added to the network. From this procedure, the result is a fully connected network of 3- and 4-node cliques (i.e., sets of connected nodes) with $3n-6$ edges. The weights in the final network are zero-order correlations.

### Walktrap Algorithm

EGA uses the Walktrap community detection algorithm (Pons & Latapy, 2005) to determine the number of *communities* (topics) in the network (Golino & Epskamp, 2017). The Walktrap algorithm uses a process known as *random walks* (or a stochastic number of edges from a certain node), which tend to get “trapped” in densely connected parts of the network. The number of steps can be specified by the user; however, EGA uses the default of 4, which has been shown to be optimal for the number of variables typically used in psychological research (Christensen, 2020; Gates, Henry, Steinley, & Fair, 2016). In the random walk process, the likelihood of a step to another node is determined by the distance between one node and all other nodes. These distances are used in an agglomerative hierarchical clustering algorithm approach, which is then subjected to merging (i.e., merging two clusters that minimize the mean of the squared distances). During the merging process, the adjacent clusters’ distances are updated to reflect the new distances between the clusters. Throughout this process, a metric to assess the quality of the partitions is used to help capture community structures at different scales. Readers interested in more detailed explanations of EGA and its components can find a detailed description in Golino et al. (2020b).

## Dynamic Exploratory Graph Analysis

Dimensionality assessment is common in psychology, but also has a significant use in data mining, especially in the subfield of text mining. Text mining is a data-driven, exploratory method used to find patterns and trends in large sets of texts, enabling the transformation of unorganized text into succinct knowledge (Ananiadou & McNaught, 2006). It is epistemologically compatible with content analysis, making it possible to collect, maintain, interpret, and discover relevant information hidden in texts in a systematic and efficient way (Singh, Hu, & Roehl, 2007). Recently, EGA was used in combination with text mining to estimate latent topics in texts, showing promising results (Kjellström & Golino, 2019).

The current implementation of EGA, however, limits its application to data collected to a single time-point (i.e., cross-sectional data). Kjellström and Golino (2019), for example, used text data from single interviews made with multiple adults about their conceptions of health. To enable the identification of latent structures in texts from social media, the EGA technique needs to be expanded to accommodate short-term temporal dynamics. This, in our view, would provide a more valid way to estimate topics in texts that are produced in a series (such as posts on Twitter). People use words to communicate their ideas, thoughts, and feelings, with groupings of words indicating the underlying content of the text (i.e., topics). If the text data come from a single time-point, the topics can be estimated using the variance–covariance matrix of the words (Kjellström & Golino, 2019). However, in the case of texts that are written on several different occasions, the temporal dynamics should be accounted for by the topic model, otherwise it may generate significant bias in the estimation of the underlying latent topics.

In texts published on Twitter, for example, people may use several words to talk about the topic of “violence” on one occasion (time or $t$). In the next occasion $(t + 1)$, the same person may use a different set of words to express their feelings about “gun control” and yet in a subsequent occasion may use different words to communicate their views about the “mainstream media.” Across time, one person can write about several different topics, using words that may or may not be the same on each occasion. Instead of modeling the covariance of words without taking time into consideration (a single-measurement approach), a more ecologically valid way to understand topics published on Twitter is to model how words are varying together across time, capturing the short-term dynamics of the texts.

One way to address this problem is proposed as follows: A collection of texts (corpus) from one single individual (e.g., a Twitter account) over $N$ discrete time-points can be represented as a document-term matrix (DTM) in which each unique word is a column and each observation (e.g., a post on Twitter) is a row in the document-term matrix. The DTM is, therefore, a $N \times K$ matrix, where $N$ is the number of time points and $K$ is the number of unique words used in the entire collection of texts. The values of the DTM cells are the frequency of the words.

Since each column of the document-term matrix represents a time series of the word frequency, $U = \left\{ u_1, u_2,\ldots , u_N\right\}$, each time series can be transformed into a time-delay embedding matrix $\mathbf {X}^{(n)}$, where $n$ is the number of embedding dimensions. A time-delay embedding matrix is used to reconstruct the attractor of a dynamical system using a single sequence of observations (Takens, 1981; Whitney, 1936). An attractor contains useful information about the dynamical system such as a series of values to which a system tends toward based on a set of starting conditions. In many empirical situations, however, the collection of possible system states (phase-space) and the equations governing the system are unknown. In such situations, attractor reconstruction techniques can be used as a means to reconstruct the phase-space dynamics using, for example, only a single time series with observable values.

In the time-delay embedding matrix, each row is a phase-space vector (Rosenstein et al., 1993):

1 $$\begin{aligned} X = \left[ X_1 X_2 \ldots X_M\right] ^\prime , \end{aligned}$$

where $X_t$ is the state of the system at discrete time $t$ and is given by:

2 $$\begin{aligned} X_t = \left[ x_t x_{t+\tau } \ldots x_{t + (n-1)\tau }\right] , \end{aligned}$$

where $\tau$ is the number of observations to offset successive embeddings (i.e., lag or reconstruction delay) and $n$ is the embedding dimension. The time-delay embedding matrix is a $M \times n$ matrix, where $M = N - (n-1)\tau$ and $N$ is the number of observations.

Suppose that $U_1$ is a column in a given document-term matrix representing the time series (of the frequency) of the word *gun*, from time $t = 1$ to $t = 10$, so that $U_1 = \left\{ 5, 6, 7,\ldots , 14 \right\}$. The frequencies of the word *gun* in this example are way beyond what one finds in textual data, especially from social media platforms, but the goal of the example is to help describe how time-delay embedding works. Transforming the time series $U_1$ into a time-delay embedding matrix with five embedding dimensions and $\tau = 1$ generates the following matrix:

3 $$\begin{aligned} \mathbf {X}^{(5)} = \begin{bmatrix} 5 &{} 6 &{} 7 &{} 8 &{} 9 \\ 6 &{} 7 &{} 8 &{} 9 &{} 10 \\ 7 &{} 8 &{} 9 &{} 10 &{} 11\\ 8 &{} 9 &{} 10 &{} 11 &{} 12\\ 9 &{} 10 &{} 11 &{} 12 &{} 13\\ 10 &{} 11 &{} 12 &{} 13 &{} 14\\ \end{bmatrix} \quad \end{aligned}$$

Once every time series of word frequency (columns) of the document-term matrix is transformed into a time-delay embedding matrix $\mathbf {X}^{(n)}$, derivatives can be estimated using generalized local linear approximation (Boker et al., 2010; Deboeck et al., 2009).

GLLA is a technique that can be used to estimate how a variable (e.g., the frequency of the word *gun*—word count per time point) changes as a function of time. The instantaneous change in one variable with respect to another variable is known as a *derivative*. The derivative can represent different aspects of change. The first derivative of a word’s time series, for example, estimates the rate of change of the word or the velocity at which the word’s frequency is changing over time. A negative first-order derivative indicates that a word is being used less often as a function of time, while a positive first-order derivative indicates that a word is being used more often as a function of time. The second derivative indicates the speed of the rate of change or the speed of how quickly a word’s frequency is changing (i.e., acceleration). A positive second-order derivative indicates an “acceleration” in the rate of change of a word’s frequency, while a negative second-order derivative indicates a deceleration in the rate of change of a word’s frequency.

In the GLLA framework (Boker et al., 2010; Deboeck et al., 2009), the derivatives are estimated as:

4 $$\begin{aligned} \mathbf {Y = \mathbf {X}L(L^{\prime }L)^{-1}}, \end{aligned}$$

where $\mathbf {Y}$ is a matrix of derivative estimates, $\mathbf {X}$ is a time-delay embedding matrix (with $n$ embedding dimensions; to simplify the notation, $\mathbf {X}=\mathbf {X}^{(n)}$), and $\mathbf {L}$ is a matrix with the weights expressing the relationship between the embedding matrix and the derivative estimates. The weight matrix $\mathbf {L}$ is a $n \times \alpha$ matrix, where $n$ is the number of embedding dimensions and $\alpha$ is the (maximum) order of the derivative. Each column of the weight matrix is estimated as follows, considering the order of the derivatives going from zero to *k*, $\alpha = [0, 1,\ldots , k]$:

5 $$\begin{aligned} \mathbf {L}_{\alpha } = \frac{[\Delta _{t}(v-\bar{v})]^{\alpha }}{\alpha !}, \end{aligned}$$

where $\Delta _{t}$ is the time between successive observations in the time series, $v$ is a vector from one to the number of embedded dimensions (i.e., $v = [1, 2,\ldots n]$), $\bar{v}$ is the mean of $v$, $\alpha$ is the order of the derivative of interest, and $\alpha !$ is the factorial of $\alpha$.

Continuing our example in which $U_1$ is a time series of the word *gun*’s frequency from time $t = 1$ to $t = 10$, we consider a time-delay embedding matrix with five dimensions, derivatives up to the second order (i.e., $\alpha = [0, 1, 2]$), and a $\Delta _{t}$ of one. This weight matrix $\mathbf {L}$ is:

6 $$\begin{aligned} \mathbf {L} = \begin{bmatrix} \frac{[1(v-\bar{v})]^{0}}{0!}, \frac{[1(v-\bar{v})]^{1}}{1!}, \frac{[1(v-\bar{v})]^{2}}{2!} \end{bmatrix} \quad = \begin{bmatrix} 1 &{} -2 &{} 2.0 \\ 1 &{} -1 &{} 0.5 \\ 1 &{} 0 &{} 0.0 \\ 1 &{} 1 &{} 0.5 \\ 1 &{} 2 &{} 2.0 \\ \end{bmatrix} \quad \end{aligned}$$

Applying Eq. 4 to estimate the derivatives, $\mathbf {Y}$ is:

7 $$\begin{aligned} \mathbf {Y} = \begin{bmatrix} 6.5 &{} 1 &{} 0 \\ 7.5 &{} 1 &{} 0 \\ 8.5 &{} 1 &{} 0 \\ 9.5 &{} 1 &{} 0 \\ 10.5 &{} 1 &{} 0 \\ 11.5 &{} 1 &{} 0 \\ 12.5 &{} 1 &{} 0 \\ \end{bmatrix} \quad \end{aligned}$$

where the first, second, and third columns represent the zeroth (observed values), first (rate of change or velocity), and second derivatives (speed of the rate of change or acceleration), respectively.

The process described above is repeated for each variable (e.g., each time series of word counts), and then, the resulting derivatives can be column bound to form a matrix $\mathbf {D}$ for each individual (e.g., each Twitter account). For instance, suppose text data from a Twitter handle *ID1* is being analyzed and the final document-term matrix has only two words (*president* and *america*) and only a few observations. Using GLLA to compute the zeroth, first, and second derivatives per word and column biding them to form a matrix $\mathbf {D}_{ ID01 }$ would result in the following matrix (Table 1).

**Table 1:** Matrix D for individual ID1

Time	ID	presid.Ord0	america.Ord0	presid.Ord1	america.Ord1	presid.Ord2	america.Ord2
1	ID01	0.34	0.49	$-$0.10	0.00	$-$0.14	$-$0.29
2	ID01	$-$0.09	0.34	$-$0.20	$-$0.10	0.29	$-$0.14
3	ID01	0.00	$-$0.09	0.00	$-$0.20	0.00	0.29
4	ID01	0.00	0.00	0.00	0.00	0.00	0.00

Using GLLA preserves both linear and nonlinear dynamics for each individual and allows different levels of analysis to be implemented. If the goal is to investigate the *population* structure, then the $\mathbf {D}$ matrices can be stacked and EGA can be used to estimate the number of underlying dimensions using data from all individuals. If the data contain multiple groups of individuals (e.g., right- and left-leaning trolls), then the $\mathbf {D}$ matrices can be stacked by group, and EGA is applied separately in each resulting stacked matrix, generating one dimensionality estimation per group. Finally, if the goal of the analysis is in the intraindividual structure, then EGA can be used in each $\mathbf {D}$. The result, in this case, will be a structure for each individual, separately. Irrespective of the level of analysis (population, group, or individual), the resulting clusters in the network correspond to variables (words) that are changing together. The main difference between EGA (Golino et al., 2020b) and DynEGA is that while the former uses the raw data to construct the network, the latter uses the *n*-order derivatives estimated via GLLA. This is, in summary, the general idea behind our Dynamic Exploratory Graph Analysis (DynEGA) approach.

One might argue that other network techniques, such as the graphical VAR network model (Epskamp et al., 2018b), could potentially be used to model the same phenomena as the DynEGA technique. Standard graphical VAR statistical methods create both a contemporaneous network of relationships between observed time series and a temporal network of discrete linear mappings from variables at time $t$ to the same set of variables at time $t + L$, where $L$ is some time lag. Nodes in these models represent variables at any given time, and the edges in these models represent either partial correlations (undirected) or average expected change between each variable at time lag $L$ (directed). What is important to note is that “change” in these models is represented solely in the estimation of the directed edges. The undirected edges do not contain information about dynamics. If, instead, nodes were created to represent how variables are changing over time (i.e., the derivatives of these variables), then the undirected edges in the contemporaneous network represent the average dynamics of change between variables and create a continuous map between each variable. Interpretations of edges between dynamic nodes in a network then represent consistent patterns of change between nodes, as opposed to value.

Undirected networks consisting of patterns of change versus value are not representing redundant information. Differences between edges in these two models may be stark. Consider two functions over time: $f(t)=t^{(-1)}$ and $g(t)=t^2$, with $t\in [1,2,\ldots ,20]$. The Pearson correlation between these two functions on this region is approximately $-.56$. However, if we take the derivative of these functions, we get $f^{\prime (t)}=-t^{(-2)}$ and $g^{\prime (t)}=2t$. The Pearson correlation between these two functions on this region is approximately $.52$, which is a change in sign from correlation of the original equations. More complex functional relationships (such as those observed in real data) may yield more differences. It is then highly likely that clustering networks based upon nodes representing change may yield valuable information above and beyond that of clustering networks based upon nodes representing values. Therefore, clustering methods based on undirected edges between raw variables (i.e., EGA) versus dynamic ones (dynEGA) can therefore lead to different findings since they are modeling different phenomena. It is important to emphasize that in the DynEGA technique, clusters of the dynamic nodes represent variables that are changing together.

The use of GLLA to estimate the derivatives in the DynEGA technique brings some challenges, especially in terms of the time-delay embedding assumption of equal time intervals between samples, and the need to set a hyperparameter in GLLA (e.g., the number of embedded dimensions) before computing the derivatives. Boker et al. (2018) investigated the impact of sampling interval misspecification in time-delay embeddings and discovered that there was no significant difference between no correction and a sophisticated full information maximum likelihood method. On the other side, setting an optimal number of embedded dimensions should impact more directly the capacity of DynEGA to estimate the topic structure since this hyperparameter governs the size of the window (i.e., number of time points) that will be used to compute the derivatives in each row of the time-delay matrix. As a consequence, this hyperparameter should be tuned using a metric of quality or fit of a multidimensional partitioning. Recently, Golino et al. (2020a) proposed a set of new fit metrics based on information theory especially designed for dimensionality assessment. In their simulation study, one of the metrics that presented the best performance (over more traditional metrics like CFI and RMSEA) was the *total entropy fit index* (TEFI), which assesses the degree of uncertainty of the partition of a multidimensional space into separate distinct categories (i.e., latent topics). Lower TEFI values indicate that a given dimensionality structure fits the data better than an alternative dimensionality solution with higher TEFI values, indicating that the former is more likely to represent the best organization of the variables. The TEFI index is calculated as follows:

8 $$\begin{aligned} \begin{aligned} TEFI_{VN} = \left[ \frac{\sum _{i=1}^{N_{F}}{\mathcal {S}(\varvec{\rho }_{i})}}{N_{F}} - \mathcal {S}(\varvec{\rho })\right] + \left[ \left( \mathcal {S}(\varvec{\rho })-\sum _{i=1}^{N_{F}}{\mathcal {S}(\varvec{\rho }_{i})}\right) \times \sqrt{N_{F}}\right] , \end{aligned} \end{aligned}$$

where $N_{F}$ is the number of topics estimated by the Walktrap algorithm, $\mathcal {S}(\varvec{\rho }_{i})$ is the Von Neumann entropy for each individual topic, and $\mathcal {S}(\varvec{\rho })$ is the total entropy of the system of variables. Golino et al. (2020a) showed that the Von Neumann entropy can be approximately estimated in a correlation matrix by scaling it so that the trace of the matrix equals one (i.e., taking a correlation matrix and dividing all entries by the number of columns of the matrix). After scaling the correlation matrix to a so-called density matrix, an entropy-like metric can be obtained by the negative of the trace of the product of the density matrix by the log of elements of the density matrix (see Golino et al., 2020a). The TEFI index can be used to tune the number of embedded dimensions in the DynEGA technique, improving the overall accuracy of our technique to estimate the number of simulated topics. The impact of tuning the number of embedded dimensions using TEFI in the capacity of DynEGA to identify the number of topics is explored in our simulation study presented in the next few sections.

### Extracting Latent Topic Trends with Topic Scores

#### Network Loadings

In these topics, certain terms contribute more information to topical trends than others. Measures to quantify the contribution of information at the nodal level (i.e., term-level) are called centrality measures. Centrality measures quantify the relative position of terms based on their connections to other nodes in the network. One of the most commonly used measures is called *node strength*, which corresponds to the (absolute) sum of a term’s connections in the network. In a series of simulation studies, Hallquist et al. (2019) demonstrated that node strength was roughly redundant with confirmatory factor analysis (CFA) loadings. They found, however, that a node’s strength represents a combination of dominant and cross-factor (cross-topic) loadings.

Considering this limitation of node strength, a more recent simulation study evaluated node strength when it’s split by dimensions (or topics) in the network (Christensen & Golino, 2021). In this study, Christensen and Golino (2021) mathematically defined a measure called *network loadings* by splitting a node’s strength based on its connections within and between dimensions identified by EGA. This measure was then standardized to derive an equivalent measure to factor loadings. Their simulation study demonstrated that network loadings can effectively estimate the population (or true) loadings and are roughly equivalent with exploratory factor analysis (EFA) loadings. They also identified effect sizes that corresponded to 0.15 (small), 0.25 (moderate), and 0.35 (large). Notably, like CFA loadings, network loadings had zeros in the loading matrix from nodes in the network that were not connected. This places network loadings on a middle ground between a “saturated” (EFA) and simple structure (CFA). Below, we provide mathematical notation for how network loadings are computed. Let $\mathbf {W}$ represent a symmetric $m \times m$ weight matrix where $m$ is the number of terms. Node strength is then defined as:

9 $$\begin{aligned} S_i = \sum _{j = 1}^n |\mathbf {W}_{ij}| \end{aligned}$$

where $|\mathbf {W}_{ij}|$ is the absolute weight (e.g., partial correlation) between node $i$ and $j$, $S_i$ is the sum of the edge weights connected to node $i$ across all nodes $n$ (i.e., node strength for node $i$). Using the definition of node strength (9), we can define node strength split between the latent topics identified by DynEGA:

10 $$\begin{aligned} \ell _{ic} = \sum _{j \in c}^C |w_{ij}| \end{aligned}$$

where $\ell _{ic}$ is the sum of edge weights in topic $c$ that are connected to node $i$ (i.e., node $i$’s loading for topic $c$), and $C$ is the number of topics (in the network). This measure can be standardized using the following formula:

11 $$\begin{aligned} \aleph _{ic} = \frac{\ell _{ic}}{\sqrt{\sum \ell _c}}, \end{aligned}$$

Note that $\sqrt{\sum \ell _c}$ is equal to the square root of the sum of all the weights for nodes in topic $c$. As pointed by Christensen and Golino (2021), the standardized network loadings ($\aleph$) are absolute weights with the signs being added after the loadings have been computed (following the same procedure as factor loadings; Comrey & Lee, 2016). In contrast to factor loadings, the network loadings are computed after the number of factors have been extracted from the network’s structure. Variables are assigned to specific factors via a community detection algorithm rather than the traditional factor analytic standard of their largest loading in the loading matrix. Therefore, some nodes may not have any connections to nodes in other topics in the network, leading some variables to have zeros for some topics in the network loading matrix. It is important to emphasize that network loadings are in the unit of association—that is, if the network consists of partial correlations, then the standardized network loadings are the partial correlation between each node and dimension.

#### Network Scores

Importantly, these network loadings form the foundation for computing network scores, which can be used to extract information about the topics in the network. Because the network loadings represent the middle ground between a “saturated” (EFA) and simple (CFA) structure, the network scores accommodate the inclusion of only the most important cross-loadings in their computation. This capitalizes on information often lost in typical CFA structures but reduces the cross-loadings of EFA structures to only the most important loadings. Below we detail the mathematical notation for computing network scores.

First, we take each topic and identify variables that do not have loadings on that topic equal to zero and divide them by the standard deviation of the corresponding variables in the data, $X$:

12 $$\begin{aligned} \varrho _{c} = \frac{\aleph _c}{\sqrt{\frac{\sum _{i=1}^{t \in c} (X_i - \bar{X})^2}{n - 1}}}, \end{aligned}$$

where the denominator, $\sqrt{\frac{\sum _{i=1}^{t \in c} (X_i - \bar{X_t})^2}{n - 1}}$, corresponds to the standard deviation of the variables (words) with nonzero network loadings in topic $c$, and $\varrho _{c}$ is the weight for the nonzero loadings in topic $c$. These weights are then made into relative weights (or proportions) and multiplied by the corresponding variables in the data, $X_{i \in c}$, to obtain the topic score:

13 $$\begin{aligned} \hat{\theta _c} = \sum \limits ^C_{c} X_{i \in c} \Bigg (\frac{\varrho _{t \in c}}{\sum ^C_c \varrho _{i \in c}}\Bigg ), \end{aligned}$$

where $\hat{\theta _c}$ is the network score for topic $c$.

It is interesting to point that one of the first researchers to discover the equivalence between network models and factor models was Guttman (1953). Although network models were not yet a specific area of research in psychology, Guttman (1953) proposed a new psychometric technique termed *“image structural analysis”*, which is basically a network model with node-wise estimation (using multiple regression). Guttman demonstrated how his new psychometric technique relates to factor models and pointed that the former is a special case of the node-wise network model where the errors of the variables are orthogonalized^1^. Therefore, it should be expected that the network scores we define here should be directly related to traditional factor scores.

## Checking the Feasibility of Dynamic Exploratory Graph Analysis to Estimate the Number of Underlying Factors/topics

Now that the DynEGA technique was described in detail, an important question must be addressed: how accurately does DynEGA estimate the number of latent topics (or latent factors). This section will briefly address this question.

A plausible underlying mechanism of latent topics can be represented as a direct autoregressive factor score model (*DAFS*; Engle & Watson, 1981), which is characterized by the autoregressive structure of the latent dimensions (Nesselroade et al., 2002). Since our paper focuses on modeling text data, we will adjust the nomenclature accordingly. In the *DAFS* framework, the observed variables $\mathbf {u}_{t}$ at time $t$ $(t = 1, 2,\ldots , N)$ are given by:

14 $$\begin{aligned} \mathbf {u}_{t} = \varvec{\Lambda }\mathbf {f}_{t}+\mathbf {e}_{t} \end{aligned}$$

where $\varvec{\Lambda }$ is the topic (factor) loading matrix (a $p \times q$ matrix), $\mathbf {f}_{t}$ is a $q \times 1$ vector of topics at time $t$, and $\mathbf {e}_{t}$ is a $p \times 1$ vector with measurement errors following a multivariate normal distribution with mean zeros and covariance matrix *Q* (Nesselroade et al., 2002; Zhang et al., 2008).

The topic scores, $\mathbf {f}_{t}$, are given by:

15 $$\begin{aligned} \mathbf {f}_{t} = \sum _{l = 1}^{L}{\mathbf {B}_{l}\mathbf {f}_{t-l}+\mathbf {v}_{t}} \end{aligned}$$

where $\mathbf {B}_{l}$ is a $q \times q$ matrix of autoregressive and cross-regressive coefficients, $\mathbf {f}_{t-l}$ is a vector of topic score $l$ occasions prior to occasion $t$, and $\mathbf {v}_{t}$ is a random shock vector (or innovation vector) following a multivariate normal distribution with mean zeros and $q \times q$ covariance matrix $\varvec{\Sigma }$ (Nesselroade et al., 2002; Zhang et al., 2008). In the *DAFS* model, $\varvec{\Lambda }$, $\mathbf {B}_{l}$, $\mathbf {Q}$ and $\varvec{\Sigma }$ are invariant over time.

Data following the *DAFS* model can be simulated using the simDFM function of the *EGAnet* package (Golino & Christensen, 2019). Below, we present a brief simulation investigating how accurately DynEGA can recover the number of simulated topics. We also investigate the distribution of variables per topic and the correlation between the simulated and the estimated topic scores. Accuracy can be calculated as follows:

16 $$\begin{aligned} ACC = \frac{\Sigma _C}{N} \text{ for } C = \Bigg \{ \begin{array}{rcl} 1 \text{ if } \hat{\theta } = \theta \\ 0 \text{ if } \hat{\theta } \ne \theta \end{array} \Bigg \} \end{aligned}$$

where $\hat{\theta }$ is the estimated number of latent topics, $\theta$ is the true number of latent topics used to simulate the data (i.e. *ground truth*), and $N$ is the number of sample data simulated.

The distribution of the variables per topic can be checked using *normalized mutual information* (NMI; Horibe, 1985). NMI is used to compare the similarity between two vectors (of discrete variables) and assigns a value of zero where the two vectors are totally dissimilar, and a value of one where they are identical in an information theoretic perspective. For example, consider two vectors ($v_1$ and $v_2$) representing the partition of a multidimensional space into two groups, so that $v_1 = (1,1,1,1,1,2,2,2,2,2)$ and $v_2 = (2,2,2,2,2,1,1,1,1,1)$. The NMI of the two vectors equals one, since both vectors are presenting the same information (i.e., that the multidimensional space is grouped in two dimensions composed by the first five elements and the last five elements, respectively). If $v_1$ or $v_2$ were to be compared to a third vector $v_3 = (1,1,1,2,2,2,2,3,3,3)$, the NMI between $v_1$ or $v_2$ and $v_3$ equals 0.38. Clearly, $v_1$ and $v_2$ exhibits the same partitioning of the multidimensional space, different from the partitioning of $v_3$.

The two vectors used to compute NMI are the vector of the *assigned variables per topic*—that generates the block diagonal matrix $\varvec{\Lambda }$ Eq. 14—and the vector containing the estimated topic number per variable.

### Simulation Design

In this brief simulation, five relevant variables were systematically manipulated using Monte Carlo methods: the number of time points (i.e., the length of the time series), topic (factor) loadings, number of variables per topic, measurement error, and type of observed data. For each of these, their levels were chosen to represent conditions that are encountered in dimensionality assessment simulation studies (e.g., Garrido et al., 2013; Golino et al., 2020b; Zhang et al., 2008) and that could produce differential levels of accuracy for DynEGA and the LDA techniques. It is important to point that most studies in the area of topic modeling do not implement Monte Carlo simulations to test the techniques and algorithms developed to estimate the number of latent topics (see Arun et al., 2010; Blei et al., 2003; Deveaud et al., 2014), using what is called *empirical evaluation* of LDA. In other words, the authors develop a new topic modeling technique (or an algorithm to decide the optimal $n$ number of topics to be extracted by LDA) and apply it to real-world text data. In the present paper, we decided to implement a brief simulation, so we could investigate how reliable is DynEGA to estimate the number of latent topics and also to compare this new technique to the widely used LDA. Therefore, the levels of the variables systematically manipulated in the current simulation were decided based on study from the area of dimensionality assessment rather than from the topic modeling literature.

For the *length of the time series*, three conditions were used: 50, 100, and 200. The number of time points was selected based on the conditions tested by Zhang et al. (2008). *Topic (factor) loadings* were simulated with the levels of 0.40, 0.55, 0.70, and 1. According to Comrey and Lee (2016), loadings of 0.40, 0.55, and 0.70 can be considered as poor, good, and excellent, respectively, thus representing a wide range of factor saturations. In addition, loadings of 1 were also simulated to allow for the evaluation of the DynEGA technique under ideal conditions.

*Number of variables per topic* were composed of 5 and 10 indicators. In the dimensionality assessment literature, there is a consensus that three variables are the minimum required for factor identification (Anderson, 1958). In the present simulation, 5 items per topic represent a slightly overidentified model, while latent structures composed of 10 variables may be considered as highly overidentified (Velicer, 1976; Widaman, 1993). The *measurement error* covariance matrix had two conditions (i.e., two diagonal matrices): one with $0.15^2$ and the other with $0.25^2$ in the diagonal. The choice to use small measurement errors was to verify how the methods perform under minimum error conditions, so the impact of the other variables systematically manipulated in our simulation could be better understood.

Two *types of observed data*, normal continuous and ordered categorical, were generated. For the ordered categorical data, a function to categorize the data based on Garrido et al. (2013) and Golino et al. (2020b) was used. First, the normal continuous data were simulated, and then, the values of the simulated observed variables were discretized to four categories. The LDA techniques were applied only to the ordered categorical data since they cannot handle normal continuous variables.

Three variables were held constant: (a) the number of topics (three), (b) the matrix with autoregressive (0.8) and cross-regressive coefficients (0), and (c) the covariance matrix for the random shock (off-diagonal = 0.18; diagonal = 0.36). The values of the autoregressive and cross-regressive coefficients and the random shock matrix were selected following Zhang et al. (2008) to ensure the topics were stationary time series (although the DynEGA model can also model non-stationary time series).

### Data Generation

For each combination of variables systematically controlled in the Monte Carlo simulation, 500 data matrices were generated according to the *DAFS* model (Eq. 14). Given the predefined parameter values, each data matrix was generated as follows. First, the matrix of random shock vectors $\mathbf {v}_{t}$ is generated following a multivariate normal distribution with mean zeros and $3 \times 3$ covariance matrix $\varvec{\Sigma }$ (off-diagonal values = 0.18; diagonal values = 0.36), where $t$ is the number of time points plus 1000 (used as the burn-in estimates for the chain). Second, the topic (factor) scores are calculated using Eq. 15 and the first 1000 estimates are removed (burn-in phase). Third, the measurement error matrix is estimated following a multivariate normal distribution with mean zeros and $p \times p$ covariance matrix $Q$, where *p* is the total number of variables (number of variables per topic times three). Finally, the observed variables are calculated using Eq. 14. Each data matrix represents data from single individuals.

### Data Analysis

The DynEGA technique was implemented using the EGAnet package (Golino & Christensen, 2019), and the following arguments of the dynEGA function were used. The number of embedding dimensions was set to five, $\tau$ (time-lag) was one and the time between successive observations for each time series was one (delta). Two network methods (GLASSO and TMFG) were used to construct the networks of the first derivatives computed using the GLLA model. The correlation between the first-order derivatives was computed using Pearson’s coefficient and was used as the input to estimate the networks.

The LDA technique was implemented via the *topicmodels* package (Hornik & Grün, 2011) using a Gibbs sampling estimator (Phan et al., 2008). Readers interested in specific details of the LDA estimation methods are referred to Hornik and Grün (2011). Since LDA requires the number of topics to be specified by the user, five approaches to estimate the optimal number of latent topics were used. The first two estimate from two to six topics and calculate the AIC and BIC of the resulting LDA solution. The remaining four approaches estimate from two to six topics and select the number of topics using the algorithms developed by Arun et al. (2010) (**Arun**), Cao et al. (2009) (**Cao**) and Deveaud et al. (2014) (**Deveaud**) using the *ldatuning* package (Nikita, 2016).

The algorithm developed by Arun et al. (2010) selects the number of latent topics that minimize the Kullback–Leibler divergence between the matrix representing word probabilities for each topic and the topic distribution within the corpus (Hou-Liu, 2018). The algorithm developed by Cao et al. (2009) selects the number of topics based on topic density, searching for the number of topics that minimizes the average cosine similarity between topic distributions, while Deveaud et al. (2014) developed an algorithm that selects the optimum number of topics by maximizing the average Jensen–Shannon distance between all pairs of topic distributions (Hou-Liu, 2018). LDA was applied in the ordered categorical data condition only, since it cannot handle continuous variables.

To investigate the impact of varying the number of embedded dimensions on the NMI and accuracy of DynEGA to recover the number of simulated topics, we also implemented a simulation in a subset of the conditions described in the *Simulation Design* section. Instead of using DynEGA with only five embedded dimensions, we used 3, 5, 7, and 9 embedded dimensions in data simulated with 100 time points (length of the time series), 5 variables per topic, measurement error covariance matrix with diagonals $0.15^2$, only three topics, and topic (factor) loadings with the following levels: .40, .55, .70, and 1. The goal is to check how varying the number of embedded dimensions impact the two performance metrics used in our paper: accuracy and normalized mutual information. We also computed the total entropy fit index for each estimated topic structure and calculated the difference in accuracy and NMI between the minimum and the maximum TEFI for all embedded dimensions used. For example, if the minimum TEFI value was obtained by the topic structure estimated using 7 embedded dimensions, and the maximum TEFI was obtained by the structure estimated using three embedded dimensions, the difference in NMI is the normalized mutual information of the former minus the NMI of the latter. A positive NMI difference means that tuning the number of embedded dimensions using TEFI is improving the allocation of items per topic (i.e., generating topics that has words closer to the population structure used in the simulation design). All codes used in the current paper are available in an online repository at the Open Science Framework platform for reproducibility purposes (see: https://osf.io/4ya6x/?view_only=b6078b404e3049818b359ae0d514f966).

### Results

#### Continuous Data

**Fig. 1:**
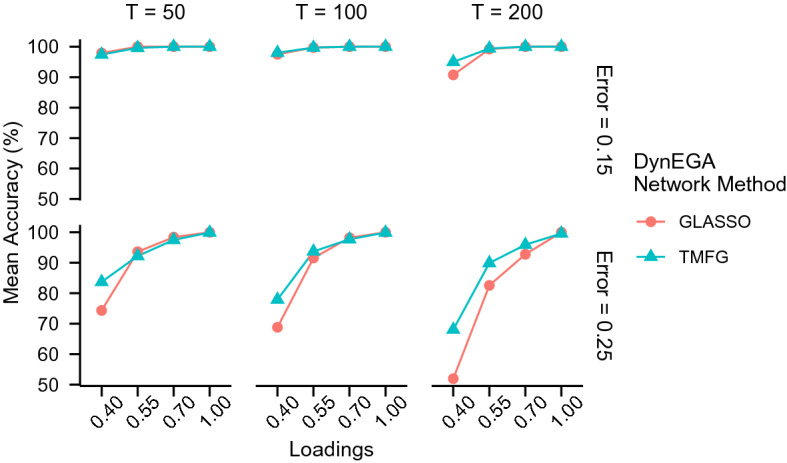
Mean accuracy per network method used in the DynEGA technique, magnitude of the loadings (*x*-axis), number of time points (vertical facets), and magnitude of measurement error (horizontal facets)

**Fig. 2:**
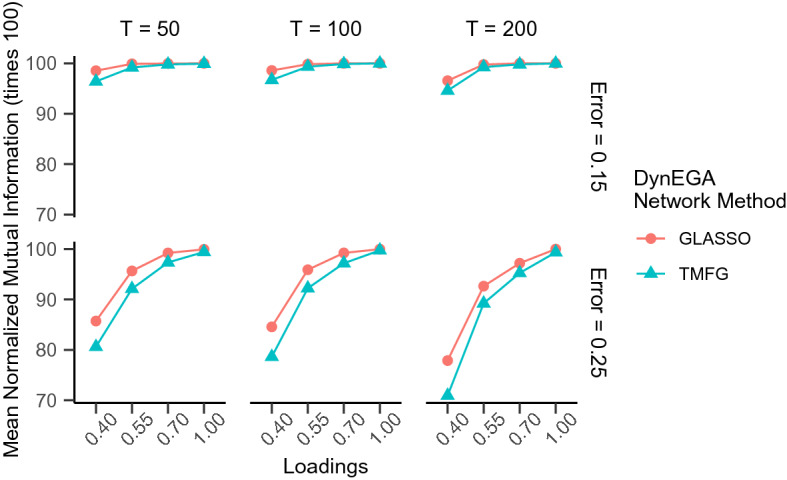
Mean normalized mutual information per network method used in the DynEGA technique, magnitude of the loadings (*x*-axis), number of time points (vertical facets), and magnitude of measurement error (horizontal facets)

In the continuous data condition, the mean accuracy of DynEGA using GLASSO ($ACC_{glasso} =$ 93.17%) and TMFG ($ACC_{TMFG} =$ 95.78%) was very similar, as were the mean normalized mutual information ($NMI_{glasso} =$ 96.62%, $NMI_{TMFG} =$ 95.38%). Figures 1 and 2 show that the mean accuracy and normalized mutual information increase with the magnitude of the loadings, but decrease with the increase in the measurement error. It is interesting to note that although the TMFG network method is more accurate (see Fig. 1), the GLASSO approach gives the higher normalized mutual information, suggesting that the latter method more accurately allocates the variables into the correct latent topics. Both figures also show that the mean accuracy and normalized mutual information decrease with the increase in the measurement error.

**Fig. 3:**
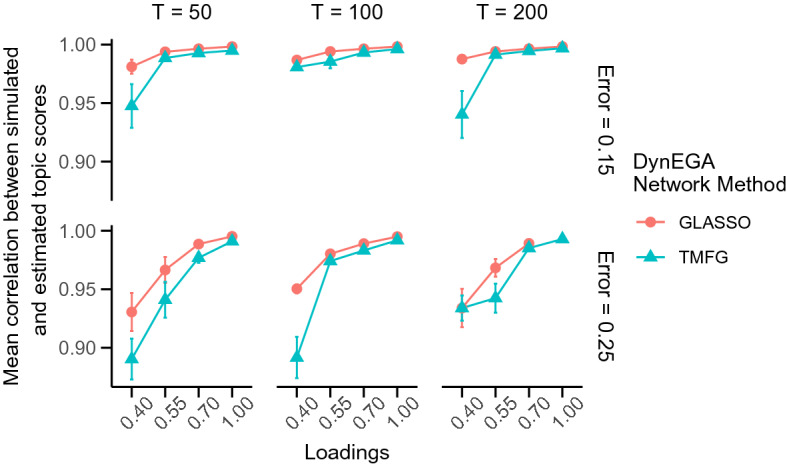
Mean correlation between simulated and estimated topic scores per network method used in the DynEGA technique, magnitude of the loadings (*x*-axis), number of time points (vertical facets), and magnitude of measurement error (horizontal facets)

Figure 3 shows the mean correlation between simulated and estimated topic scores per network method used in the DynEGA technique, magnitude of the loadings, number of time points and magnitude of the measurement error. The DynEGA technique with the GLASSO network method presented a higher mean correlation (GLASSO) than the TMFG network method (TMFG). The differences are higher for factor loadings of 0.40 (see Fig. 3).

#### Ordered Categorical Data

In the ordered categorical data condition, the scenario is slightly different. The mean accuracy of DynEGA using the TMFG network method ($ACC_{TMFG} = 85.70\%$) was higher than the mean accuracy of GLASSO ($ACC_{glasso} = 73.43\%$). As with the continuous data type, DynEGA with the GLASSO network method presented a higher mean normalized mutual information than DynEGA with TMFG ($NMI_{glasso} = 88.20\%$, $NMI_{TMFG} = 85.67\%$).

The strategies used to select the optimal number of topics via LDA all presented a very low accuracy. The *Arun* algorithm (Arun et al., 2010) had an accuracy of zero since it always selected the maximum number of topics compared (six) as the optimal number of topics. AIC and BIC presented a mean accuracy of 14.62% and 16.84%, respectively. *Cao’s* (Cao et al., 2009) and *Deveaud*’s algorithm (Deveaud et al., 2014) presented the best mean accuracy: 28.60% and 31.24%, respectively. Figure 3 shows the mean accuracy per method in the ordered categorical data condition, per loadings (*x*-axis), number of time points (vertical facets), and magnitude of measurement error (horizontal facets). The DynEGA technique with the TMFG network method had the highest accuracy, followed by DynEGA with the GLASSO, and the *Deveaud*’s algorithm and *Cao*’s algorithm for LDA.

**Fig. 4:**
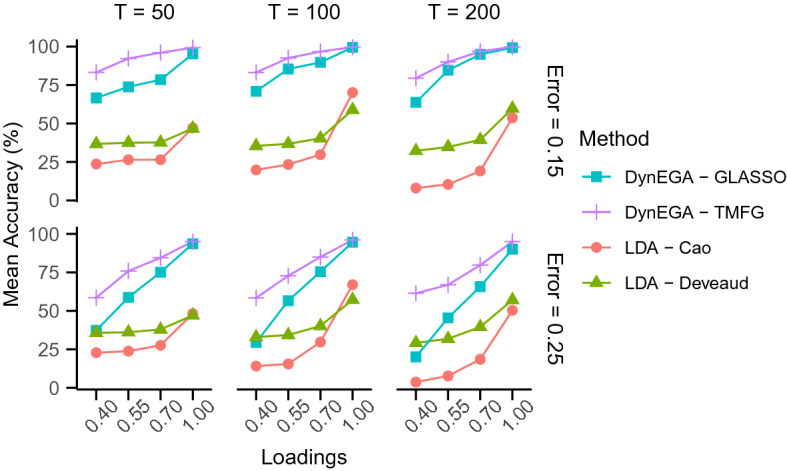
Mean accuracy per method in the ordered categorical data condition. Magnitude of the loadings (x-axis), number of time points (vertical facets), and magnitude of measurement error (horizontal facets)

**Fig. 5:**
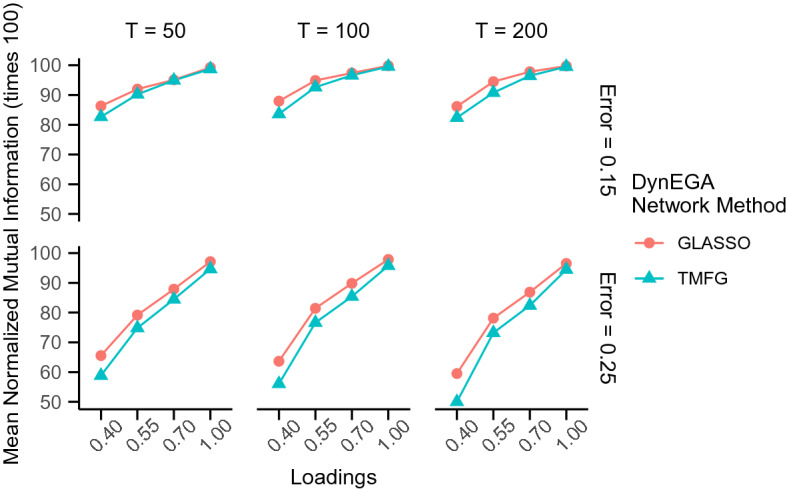
Mean normalized mutual information per network method used in the DynEGA technique, magnitude of the loadings (*x*-axis), number of time points (vertical facets), and magnitude of measurement error (horizontal facets)

Figure 5 shows that the DynEGA with the GLASSO network method presented a slightly higher mean normalized mutual information than the DynEGA with the TMFG network method, specially with loadings of 0.40, 0.55 and 0.70.

**Fig. 6:**
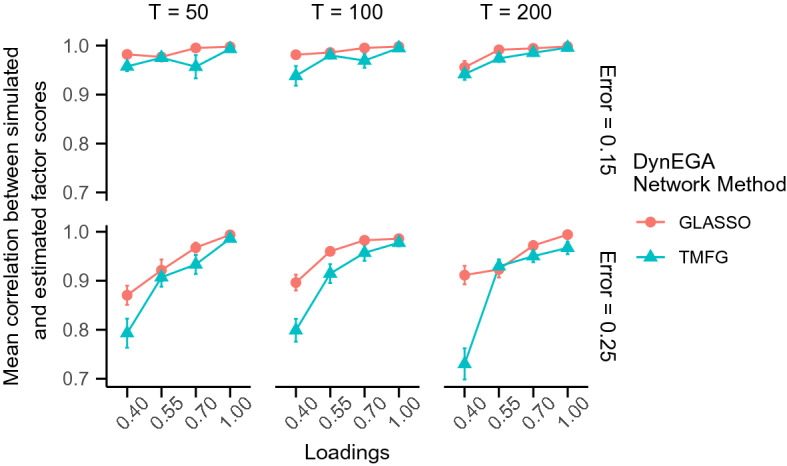
Mean correlation between simulated and estimated topic scores for the ordered categorical data condition per network method used in the DynEGA technique, magnitude of the loadings (*x*-axis), number of time points (vertical facets), and magnitude of measurement error (horizontal facets)

Figure 6 shows the mean correlation between simulated and estimated topic scores per network method used in the DynEGA technique, magnitude of the loadings, number of time points and magnitude of the measurement error. The DynEGA technique with the GLASSO network method presented a higher mean correlation between the simulated and estimated topic scores (GLASSO) than the TMFG network method (TMFG). The differences are higher for loadings of 0.40 (see Fig. 6).

#### Varying the number of embedded dimensions

As pointed before, the number of embedded dimensions used to compute the derivatives in the GLLA technique can impact the accuracy of DynEGA in the estimation of the number of simulated topics. We used a subset of the conditions implemented in the simulation above to investigate how setting the number of embedded dimensions to 3, 5, 7 and 9 impacted the NMI and accuracy of DynEGA in data simulated with 100 time points (length of the time series), 5 variables per topic, measurement error covariance matrix with diagonals $0.15^2$, only three topics, and topic (factor) loadings with the following levels: .40, .55, .70, and 1.

**Fig. 7:**
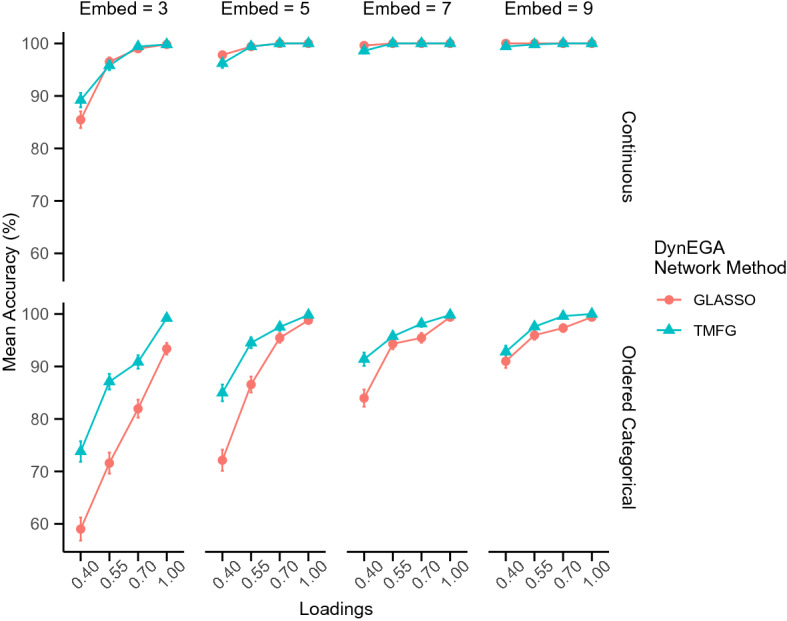
Mean accuracy per number of embedded dimensions, method, magnitude of the loadings (*x*-axis), number of embedded dimensions (vertical facets), and data type (horizontal facets)

Figure 7 shows the mean accuracy for each DynEGA network method, magnitude of the loadings, number of embedded dimensions and data type. It is interesting to note that the mean accuracy increases with the number of embedded dimensions, with an overall accuracy of 95.90% for three embedded dimensions, 99.10% for five, 99.78% for seven, and 99.90% for nine embedded dimensions. The impact of varying the number of embedded dimensions is higher for ordered categorical data, and for moderate and low factor/topic loadings. For example, the mean accuracy of DynEGA with the GLASSO network technique when the factor/topic loadings is 0.40 goes from 59.02% with three embedded dimensions to 91% with nine embedded dimensions for the ordered categorical data.

**Fig. 8:**
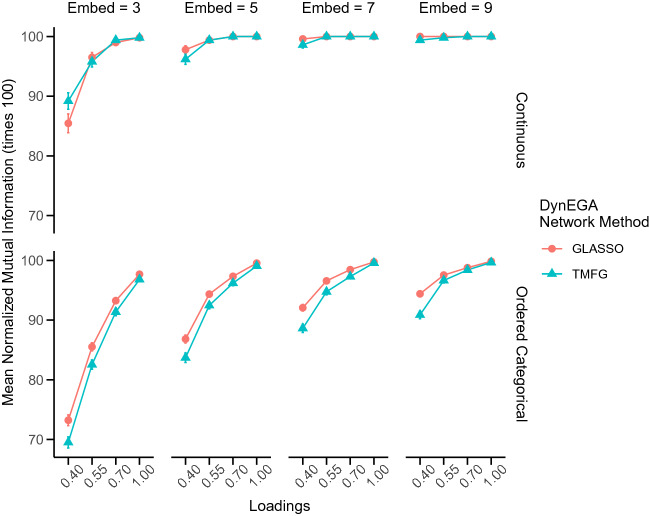
Mean normalized mutual information per number of embedded dimensions, method, magnitude of the loadings (*x*-axis), number of embedded dimensions (vertical facets), and data type (horizontal facets)

A similar pattern happens with the normalized mutual information. Figure 8 shows the mean normalized mutual information for each DynEGA network method, magnitude of the loadings, number of embedded dimensions and data type. The mean NMI increases with the number of embedded dimensions, with an overall accuracy of 95.9% for three embedded dimensions, 99.1% for five, 99.78% for seven, and 99.9% for nine embedded dimensions. The impact of varying the number of embedded dimensions is higher for ordered categorical data, and for moderate and low factor/topic loadings. For example, the mean accuracy of DynEGA with the GLASSO network technique when the factor/topic loadings is 0.40 goes from 73.22% with three embedded dimensions to 94.40% with nine embedded dimensions for the ordered categorical data.

Using the total entropy fit index (TEFI) to tune the number of embedded dimensions allows the researcher to set this hyperparameter of the DynEGA technique in an objective way. The TEFI is a relative measure of fit, whose value is lower for structures that present a better fit to the data (Golino et al., 2020a). When the TEFI index is computed for the topic structures estimated using DynEGA with three, five, seven, and nine embedded dimensions, a decision can be made in terms of which number of embedded dimensions leads to the structure that best fits the data.

**Fig. 9:**
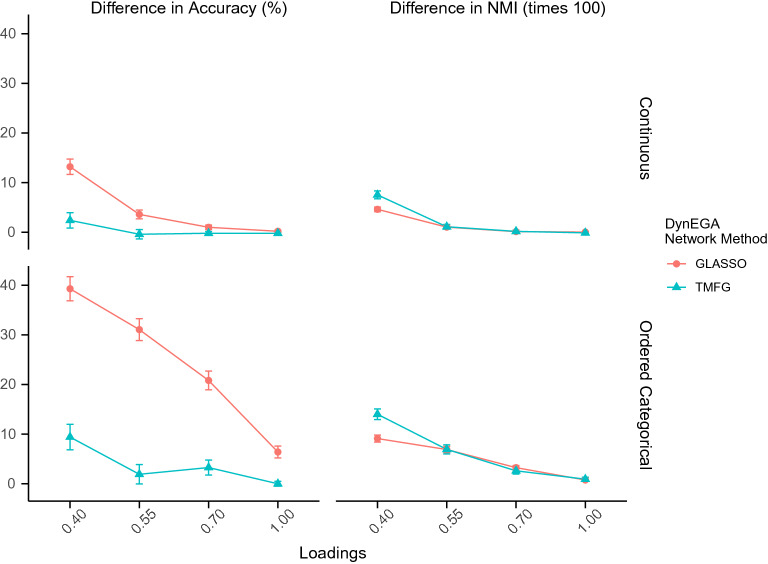
Mean difference in accuracy and normalized mutual information between the structure with the lowest TEFI value and the maximum TEFI value per magnitude of the loadings (*x*-axis), network construction method (colors), and data type (horizontal facets)

Figure 9 shows the difference in accuracy and normalized mutual information between the structure with the lowest TEFI value and the structure with the maximum TEFI value per magnitude of the loadings (*x*-axis), network construction method (colors), and data type (horizontal facets). Therefore, the *y*-axis of Fig. 9 represents gain in accuracy and NMI obtained by tuning the number of embedded dimensions using the TEFI index, which is larger for the ordered categorical data than for the continuous data. A trend worth noting is that tuning the number of embedded dimensions leads to higher gains in conditions with more error due to lower topic (factor) loadings. In terms of gains in accuracy, DynEGA with the GLASSO network method benefited more than the TMFG network method, gaining 13.20% in the continuous data condition and 39.29% in the ordered categorical data condition for loadings of .40. The gains in accuracy and in NMI fade away with the increase in the topic (factor) loading since with higher loadings DynEGA is, in general, very accurate to recover the number and composition (i.e., correct placement of words per topic) of the simulated topic structure. Here, DynEGA with TMFG benefited more in terms of NMI than DynEGA with GLASSO.

## Applying the Dynamic Exploratory Graph Analysis in the IRA-linked Twitter Data

The substantive problem this paper addresses is related to the use of social media, especially Twitter, by agencies or groups of people devoted to exploit social divisions in a society for political reasons. People are increasingly using online social platforms to communicate their ideas, and politicians and government bodies are taking advantage of these platforms to leverage their interaction with voters and citizens. Social media platforms are becoming more and more central in debates about relevant issues such as abortion, gun control, and other controversial topics (Rajadesingan & Liu, 2014), sometimes leading to strongly polarized positions (Yardi & Boyd, 2010).

In the USA, online activities were used to influence public opinion and voters during the 2016 campaign, with attacks occurring before and during the electoral process (Linvill & Warren, 2018). Twitter accounts linked to the Internet Research Agency (IRA), based in Russia, were used to try to divide voters in a wide range of issues (Linvill & Warren, 2018), and their information was released by the US congress after an investigation to discover Russian state-sponsored trolls (Zannettou, Caulfield, De Cristofaro, et al., 2019a). In this section, the DynEGA technique is applied to a large database of IRA-linked Twitter accounts extracted by Linvill and Warren (2018) and posted online by the FiveThirtyEight team (Roeder, 2018). The goal is to investigate the strategies used by right- and left-wing Twitter accounts.

The original data contain almost 3 million Twitter posts, by 2843 unique accounts, starting in January 2013 to May 2018. Linvill and Warren (2018) classified the accounts into five groups: right troll, left troll, news feed, hashtag gamer, and fearmonger. The first two types of accounts mimic right- or left-leaning people. The news feed accounts present themselves as local news aggregators, the hashtag gamer accounts specialize in playing hashtag games, and the fearmonger accounts spread news about a fake crisis (Roeder, 2018). In the current analysis, only accounts identified as right- and left-leaning trolls were used, with posts (not including retweets) from January 2016 to January 2017. Accounts with less than 50 posts were excluded, resulting in 276,752 posts (78.32% from left-leaning trolls) and 236 accounts. The 236 accounts included in the analysis can be considered influential, since they have a nonnegligible number of followers (median = 877, mean = 4838), with 12.37% having more than 10,000 followers. The high number of followers can help the trolls in pushing specific narratives to a much greater number of Twitter users (Zannettou et al., 2019a).

An omnibus analysis was implemented using data from both types of trolls (i.e., right and left-leaning trolls) that were pre-processed using the *tm* package from R (Feinerer et al., 2008). URLs were removed from the text data as well as punctuation, numbers, and stop words. All characters were converted to lowercase, and the words were stemmed (i.e., reduced to their stem, base, or root using *Porter’s algorithm*; Porter, 1980). The sparsity of the resulting document term matrix (i.e., a data frame where the columns are unique words and rows are different documents or posts) was decreased using a threshold of .9935, so that words with a sparsity above the threshold are removed from the document term matrix, resulting in 170 unique words for the right and 113 words for the left trolls. In text mining, deciding the number of words to use is done arbitrarily. In the current analysis, we decided to use between 100 and 200 words, so the final document-term matrix would have enough words to capture different types of topics, but not very specific topics (or niche content), which could happen if the number of words used increases (since the number of words used depends on the sparsity threshold, using more words means using words that are less frequently used).

To estimate the topics per account type, the DynEGA technique was used via the dynEGA function from the EGAnet package. Since the TMFG network method presented the highest accuracy in the estimation of the number of topics in the ordered categorical data, it was the network estimation method used in the current analysis. The optimum number of embedded dimensions was tuned using the procedure presented in our simulation study: different numbers of embedded dimensions were used (5, 10, 15, and 20), and the fit of the resulting topic structure estimated by DynEGA was verified using the total entropy fit index. The number of embedded dimensions associated with the lowest TEFI value was selected and referred to as $\kappa _n$, being $\kappa$ the TEFI index and *n* the number of embedded dimensions. The TEFI values per number of embedded dimensions were as follows: $\kappa _{(5)} = -108.00$, $\kappa _{(10)} = -113.38$, $\kappa _{(15)} = -107.03$, and $\kappa _{(20)} = -109.48$, and therefore, the number of embedded dimensions was set to 10. The other dynEGA arguments were set as follows: $\tau$ (time-lag) was set to one and the time between successive observations for each time series was one (delta). The correlation between the first-order derivatives was computed using Pearson’s coefficient and was used as the input to estimate the network. The level of analysis used in this section was set to *population*, meaning that the derivatives are estimated per Twitter account and then row binded, creating a long data frame that is used to compute the correlation matrix. The estimated topics are, then, the “mean” structure of population.

**Fig. 10:**
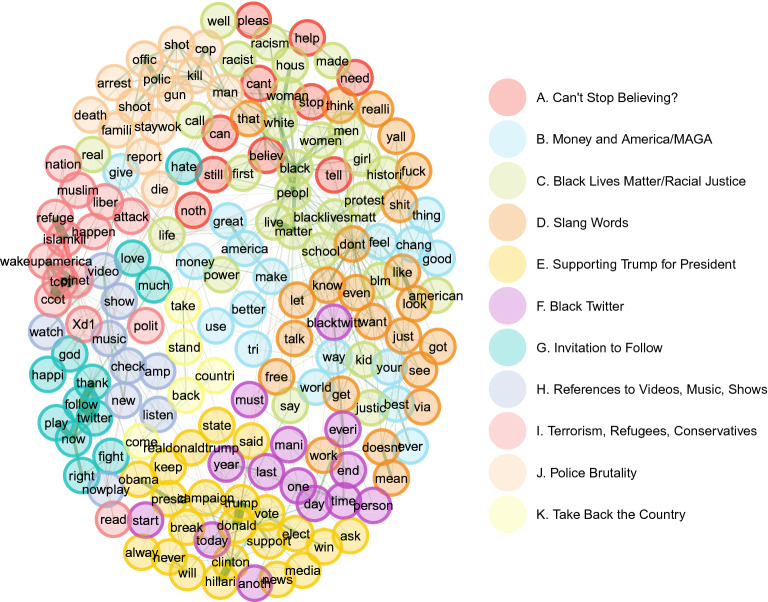
Network structure estimated using DynEGA of the right trolls document-term matrix showing eight topics (clusters). The nodes represent the words and the edges are the Pearson correlation of the words’ first-order derivatives

Figure 10 shows the network structure of the left- and right-leaning trolls Twitter data (170 words). The nodes represent the words and the edges are the Pearson correlation of the words’ first-order derivatives. Eleven topics were estimated. The first topic (topic A in Fig. 10) was one of the least interpretable topics, with words such as *can’t*, *stop*, *believe*. The second topic (topic B in Fig. 10) makes reference to money and Donald Trump’s campaign slogan (*Make America Great Again* or MAGA). The third topic (topic C in Fig. 10) makes reference to the *Black Lives Matter* movement, racism, and racial justice. The fourth topic (topic D in Fig. 10) is a small collection of slang words and cursing, and the fifth topic (topic E in Fig. 10) seems to demonstrate support for Trump running for president, make references to former President Obama and former Secretary of State Hillary Clinton (also running for President in 2016), and to the media. The sixth topic (topic F in Fig. 10) emphasizes the *Black Twitter* activities, while the seventh topic (topic G in Fig. 10) seems to invite people to follow users and/or topics (example of words: *follow*, *thanks*, *now*, *twitter*). The eighth topic (topic H in Fig. 10) has words as *listen*, *video*, *show*, *now playing*, *watch*, making references to live streaming of music and video content. The ninth topic (topic I in Fig. 10) makes reference to terrorism (using expressions as *terrorism* and the hashtag *islamkill*), refugees, the conservative movement in America (e.g., the abbreviations *PJNET*, *CCOT* and *TCOT* are referencing the *Patriot Journalism Network*, *conservatives on Twitter*, and *Top Conservatives on Twitter*) and the *wake up America* movement (a call for conservative principles endorsed by Donald Trump and others). The tenth topic (topic J in Fig. 10) makes reference to reports of police brutality and the eleventh topic (topic K in Fig. 10) talks about *taking back the country*, also a slogan used by pro-Trump supporters.

**Fig. 11:**
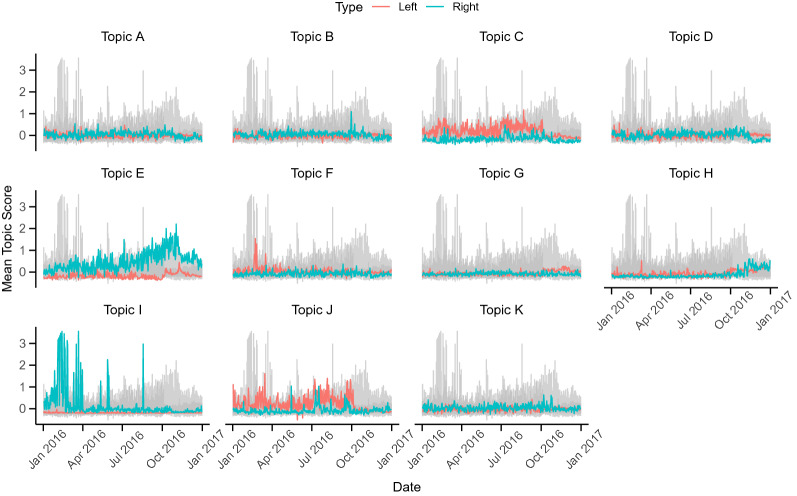
Latent trends of the topics per date

**Fig. 12:**
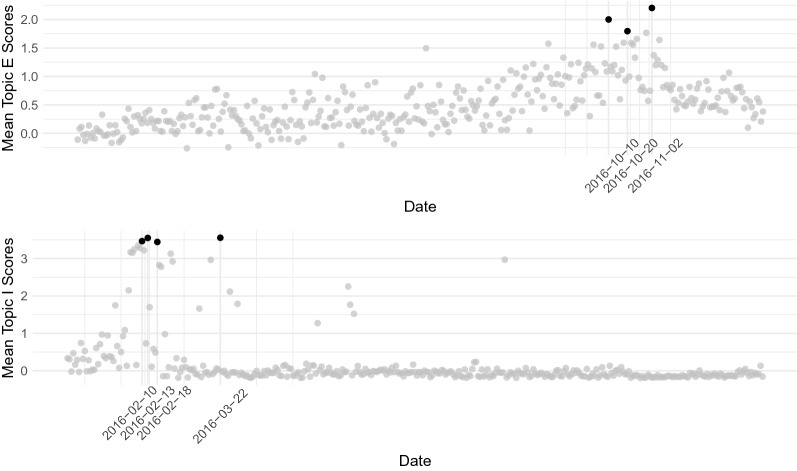
Latent trends of topic E (supporting Trump for President) and topic I (Terrorism, Refugees, Conservatives)

To understand how each topic is expressed over time, the topic scores are estimated using Eq. 13. Figure 11 shows the mean topic score per date by type of trolls (left- and right-leaning trolls). Topics C (*black lives matter*), F (*black twitter*), H (*references to music, videos and shows*) and J (*police brutality*) are used more often by the left-leaning trolls, while topics E (*supporting Trump for president*), I (*terrorism, refugees, conservatives*) and K (*take back the country*) are more usually employed by the right-leaning trolls. Together, these topics (C, E, F, H, I, J and K) represent content more related to the political agenda of pro-Trump movements or more liberal, anti-racism groups in the USA. Some trends are interesting to mention. For example, topic E (*supporting Trump for president*) peaked three times, once in October 10th (the day after the second presidential debate, in which major news agencies started transcribing and analyzing all the controversial rhetoric of Donald Trump), once in October 20th (date of Trump’s rally in Delaware, in which he said that he would accept the results of the elections if he was the candidate elected) and once in November 2nd, 2016 (date in which he had multiple rallies in Florida for thousands of people). Topic I (*Terrorism, Refugees, Conservatives*) presented peaks on March 22nd (day of the bombings at Brussels airport and a metro station that killed 32 people) and in three days of February (02/10, 02/13, and 02/18), dates in which reports about the immigration crisis in Europe surfaced and were reported by major news outlet. Specifically on February 18, 2016, the Department of Homeland Security announced further travel restrictions for travelers that visited Libya, Somalia, and Yemen. The examples for topics E and I are presented in Fig. 12.

**Fig. 13:**
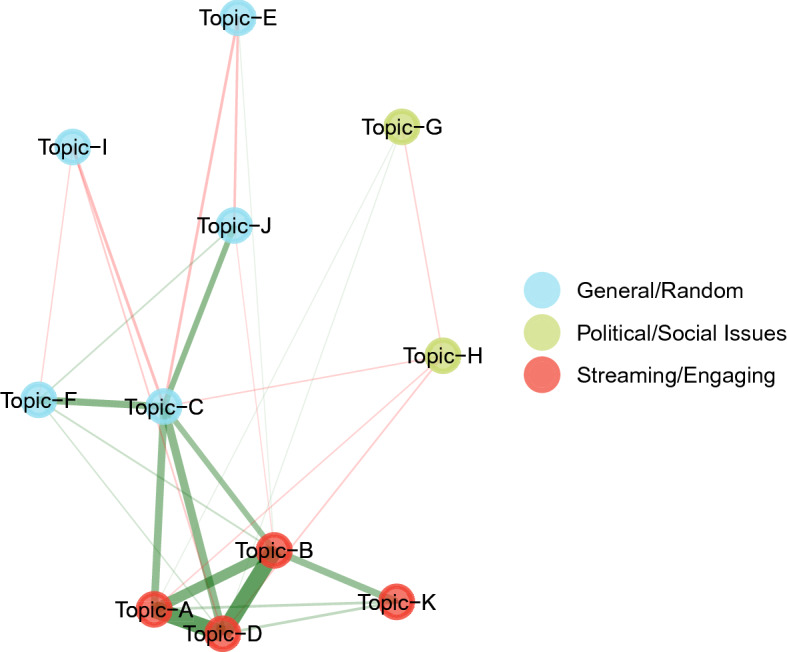
Network structure estimated using DynEGA of the first-order topics. The nodes represent the network scores for each topic, and the edges are the Pearson correlation of scores’ first-order derivatives

The estimated topic scores can be used to estimate a second-order topic structure (similar to second-order factors), in which first-order topics can be grouped into second-order broad *themes*. To do that, DynEGA is used as before (i.e., tuning the number of embedded dimensions using the TEFI index), but instead of using the raw document-term matrix to estimate the first-order derivatives, the estimated topic scores can be used instead. Six values for the embedded dimensions were used (5, 10, 15, 20, 25, and 30), and the resulting TEFI values are: $\kappa _{(5)} = -5.02$, $\kappa _{(10)} = -3.53$, $\kappa _{(15)} = -3.59$, $\kappa _{(20)} = -5.66$, $\kappa _{(25)} = -5.63$, and $\kappa _{(30)} = -5.63$. Therefore, the number of embedded dimensions was set to 20 (minimum TEFI value). The other dynEGA arguments were set as before, with a time-lag ($\tau$) and time between successive observations for each time series was both set to one (delta). Again, the correlation between the first-order derivatives was computed using Pearson’s coefficient and was used as the input to estimate the network, and the level of analysis was set to *population* (meaning that the derivatives are estimated per Twitter account and then row binded, creating a long data frame that is used to compute the correlation matrix). The resulting network of the first-order topics and the estimated second-order *themes* can be seen in Fig. 13. Three broad *themes* were estimated. One is a combination of political and social issues topics (C, E, F, H, I, J and K), while the second is a combination of general topics (B and K) and random ones (A and D). The third general *theme* is composed by topics inviting people to follow an account or twitter discussion (topic G) and mentioning music, videos, shows, and live streaming content (topic H). It is worth noting that within *theme* covering political and social issues content, the topics that are more common in the left-leaning trolls (topics C, F, and J) present a positive association among themselves and a negative association with topics E and I (more common in the right-leaning trolls). This negative association might be an indication that there is some underlying principle guiding the posts from left- and right-leaning trolls on Twitter.

To check what type of trolls is leading (or forcing/driving) the posts of political/social issues themes on Twitter, we used convergent cross-mapping (Clark et al., 2015; Sugihara et al., 2012)—a technique used to identify the coupling of time-series. If none types of trolls are driving the other, then convergent cross-mapping will lead to a nonsignificant “causal” signal, computed using the bootstrapped 95% confidence intervals for the estimated Pearson correlation coefficient of estimates of the time series of one group (time-series A) from the time series of the another group (time-series B) for each number observations tested (for more details, see Clark et al., 2015). The convergent cross-mapping estimated a nonsignificant coupling in which the left trolls drive the right trolls ($p = 0.4254$), and a small, but nonsignificant, *p* for the right-leaning trolls driving the left-leaning trolls ($p = 0.05$). The small *p* value for the convergent cross-mapping in which the right-leaning trolls are driving the left-leaning trolls instigated us to explore the data in a more granular way, using the network scores from the first-order topics instead of the scores of the second-order *theme* with the political/social issues content. Since topics C (*Black Lives Matter/Racial Justice*) and I (*Terrorism, Refugees and Conservatives*) were among the two most frequent topics for the left- and the right-leaning trolls, respectively, we choose to focus on these two time series (i.e., the mean network score per date of topic C for the left and topic I for the right-leaning trolls). The convergent cross-mapping showed a nonsignificant coupling in which the topic C (*Black Lives Matter/Racial Justice*) for the left-leaning trolls drives topic I for the right-leaning trolls ($p = 0.074$), but a significant coupling in which topic I for the right-leaning trolls drives or forces topic C for the left-leaning trolls ($p = 0.014$). Convergent cross-mapping did not detect a significant coupling in neither direction for topics C and E (*Supporting Trump for president*).

## Discussion

Quantitative analysis of texts can be very useful for understanding the strategies used in online information warfare. In the past few years, a number of studies investigated characteristics of Twitter accounts created to sow discord and polarization before, during, and after the 2016 presidential election in the USA (Ghanem et al., 2019; Llewellyn et al., 2018; Zannettou et al., 2019a; Zannettou et al., 2019b). A common denominator in these studies has been the use of latent Dirichlet allocation (LDA; Blei et al., 2003) to estimate latent topics from posts on social media platforms. As pointed out earlier, despite the usefulness of LDA, it has several limitations that make its use in text data from social media platforms questionable.

The estimation of the number of latent topics is a problem akin to the dimensionality assessment problem in psychometrics. Although some algorithms were developed to check the optimal number of latent topics estimated via LDA (Arun et al., 2010; Cao et al., 2009; Deveaud et al., 2014), their implementation involves multiple steps and was not widely available in software until very recently (Nikita, 2019), which may help explain why researchers choose the number of topics in LDA arbitrarily.

In terms of interpretability, topic modeling methods are statistical tools in which numerical distributions must be explored to generate a meaningful interpretation of the results (Chaney & Blei, 2012). In LDA and most topic modeling techniques, the output of the models does not provide enough information to generate a straightforward interpretation of the latent topics (Chaney & Blei, 2012), and the researchers must check the distribution of word probabilities per topic in order to make sense of them. In real-world data, interpreting the distribution of word probabilities per latent topic is not just cumbersome, but sometimes the words with the highest probabilities in each topic are very similar, making the interpretation of the content of the topics in LDA (and other topic modeling methods) a challenge.

In the current paper, we introduced a new method to estimate latent dimensions (e.g., factors, topics) in multivariate time-series data termed Dynamic Exploratory Graph Analysis. The DynEGA technique can be used to estimate the latent structure of topics published in social media (using the time series of word frequencies), improving our capacity to understand the strategies used by accounts created as tools of information warfare. Unlike LDA, DynEGA automatically identifies the number of topics and the distribution of variables (words) per topic and can model temporal dynamics in both stationary and non-stationary time series (although the simulation only focused on stationary time series). Network loadings and network scores can be computed from the DynEGA results, opening up the possibility of estimating second-order dimensions (e.g., second-order *themes*), and reducing the dimensionality of the data from multiple time series of words to a handful of dynamic themes. A brief Monte Carlo simulation study was implemented to check the capacity of DynEGA to recover the parameters (i.e., number of topics and topic scores) used to simulate the data using the direct autoregressive factor score model (Engle & Watson, 1981; Nesselroade et al., 2002).

The results showed that the DynEGA technique presented a very high performance in recovering the number of simulated topics, especially when the variables have moderate and high loadings (for both the continuous and ordered categorical data conditions). DynEGA with the TMFG network method presented a higher accuracy in estimating the number of simulated topics compared to the GLASSO network method for both types of variables (continuous and ordered categorical), and is the technique we recommend for topic modeling. However, it is important to point that DynEGA with GLASSO presented a slightly higher normalized mutual information and correlation between the simulated and the estimated topic scores.

DynEGA presented an accuracy considerable higher than LDA in the ordered categorical data condition. Of the LDA techniques, *Cao’s* (Cao et al., 2009) and *Deveaud*’s algorithms (Deveaud et al., 2014) presented the best mean accuracy among the other LDA techniques, but never exceeded the 75% accuracy threshold (Fig. 4). By comparison, DynEGA with the TMFG network method was at or above the 75% accuracy threshold in most of the conditions tested (Fig. 4). The results of our Monte Carlo simulation present evidence that LDA should be used with caution when applied in data from social media platforms such as Twitter. However, more simulation studies are needed to verify the conditions in which LDA works well and the conditions it fails. At the same time, the new technique presented in this paper shows a high accuracy in estimating the number of simulated topics and a high normalized mutual information for the moderate- and high-loading condition. Moreover, the DynEGA methods had a very high correlation between the simulated and the estimated topic (factor) scores (Fig. 6), irrespective of the condition tested.

Since DynEGA uses generalized local linear approximation (Boker et al., 2010), we also investigated the impact of varying the number of embedded dimensions used to compute the derivatives (a hyperparameter of GLLA) in the accuracy of our technique to estimate the number of simulated topics. We discovered that as we increased the number of embedded dimensions, both the accuracy and the normalized mutual information improved, especially for low and moderate loadings. Therefore, tuning this hyperparameter is important before applying the DynEGA technique, and we proposed the use of the total entropy fit index as a measure of fit for the estimated topic structure. We also showed in our simulation that optimizing or tuning the number of embedded dimensions using TEFI substantially improved the accuracy and normalized mutual information, especially in ordered categorical data, moderate and low loadings, and for the GLASSO network method. Future research should also investigate the impact of using different values for the $\tau$ (time lag) parameter in the accuracy of the DynEGA technique to recover the number of simulated dimensions (topics or factors).

To exemplify the use of DynEGA, we applied it to a large database of IRA-linked Twitter accounts containing right- and left-leaning trolls (Linvill & Warren, 2018). The results revealed a very interesting set of online information warfare strategies. The right-leaning trolls were posting content supporting Donald Trump’s presidential campaign and promoting the conservative movement in the US, defending a political agenda aligned with Trump’s *Make America Great Again* movement, and posting content related to refugees, anti-terrorism, and anti-Islam. The left-leaning trolls were posting content supporting the Black Lives Matter movement, denouncing police brutality, promoting the *black Twitter*, and promoting content related to music, videos, and shows. Notably, the trolls associated with the Internet Research Agency seemed to follow the news coverage of the 2016 US electoral very closely as well as local and international events that could be used to promote their political agenda. Using DynEGA topic scores, we discovered that the right-wing trolls were posting more topic-related content than usual on important dates, coinciding with: (1) the second presidential debate in 2016, (2) Trump’s rally in Delaware, in which he said that he would accept the results of the elections if he was the candidate elected, (3) the day of bombings at Brussels airport and a metro station, and (4) dates in which reports about the immigration crisis in Europe surfaced and were reported by major news outlet.

Rather than only spreading content to advance their political agenda, the IRA-linked trolls were following a strategy to amplify important events in the USA. This strategy may serve two different goals: disguise themselves as regular citizens and to use daily news events to promote a pre-defined, state-sponsored agenda (Linvill & Warren, 2018; Zannettou, Caulfield, De Cristofaro, et al., 2019a). The content posted by the IRA accounts had a very clear strategy to promote pro-Trump and a pro-conservative agenda (proliferated by the right-leaning trolls) as well as discussions about the *black lives movement*, racism, racial justice, and related topics, in which the left-leaning trolls were in charge of, meaning that the right- and left-wing trolls differed substantially in terms of content and targeted a specific audience, in line with the previous research (Ghanem et al., 2019; Stewart et al., 2018). This indicates that the minds behind the online intelligence (or information warfare) operation targeted content that could potentially maximize the activation (in terms of online interactions) of a network of Twitter users that shared similar political views and that could be more prone to engage in more extreme political activities online and offline (Fenton, 2016). These strategies might have been designed to impact the debate on social media about the 2016 US elections, in an attempt to generate political and opinion polarization, which increases when opinions becomes “dispersed, bimodal, closely associated and closely linked to social identities” (Grover, Kar et al., 2019, p. 440).

The topic scores computed from the DynEGA results were used to estimate a second-order structure of *themes*, similar to second-order factors. Three themes were identified, with the most relevant one composed of topics focusing on politics and social issues. Topics that are more frequent for the left-leaning trolls presented a negative association with topics that are usually employed by the right-leaning trolls. Using a convergent cross-mapping analysis, we discovered that right-leaning trolls, despite having a smaller percentage of posts (only 28% of the total posts used in our analysis), were the force driving the posts in the general area of politics and social issues made by the left-leaning trolls. In a more granular analysis, we discovered that the topic focusing on terrorism, refugees, and the conservative movement in the USA was driving the left-leaning posts in the topic related to the *black lives matter* movement. This seems to be an additional evidence that the masterminds of the online intelligence operation wanted to maximize polarization in Twitter, by promoting two opposing political agendas, but in a way that is organized with a specific directionality: the right-leaning trolls lead the political and social issues debate, and the left-leaning trolls follow by disseminating their specific agenda.

Our analysis revealed topics that were pertinent to several consequential events of the election cycle, demonstrating the coordinated effort of trolls capitalizing on current events in the USA. This demonstrates the potential power of our approach for revealing temporally relevant information from qualitative text data. Such an approach has applications that extend far beyond the election cycle. Twitter, for example, contains all sorts of information related to people’s preferences and patterns of behavior that may be relevant for other data mining endeavors such as advancing our understanding of idiographic personality (e.g., Bleidorn & Hopwood, 2019). Outside of Twitter, DynEGA opens up opportunities for quantifying daily diaries and essays in a meaningfully and concise way. One example might be extracting the underlying affective information in daily diaries of clinical samples to determine whether certain people are responding to treatment or are prone to remission. DynEGA further supports analyses across levels—from individuals to groups to population—which opens up avenues for evaluating the connections between within- and between-person structures, which is a timely topic of interest in a number of areas in psychology (e.g., personality; Baumert et al., 2017).

The dynamic modeling of texts is an increasingly important topic in psychology because of the amount of information that’s stored in qualitative data. Whether its over social media or qualitative coding of ecological behaviors, effectively extracting quantitative information that can be leveraged to make predictions about people’s behavior is underdeveloped area in research. Here, we provide a tool that can quantify this information in a meaningful way so that researchers can identify latent and qualitative manifestations of behavior. DynEGA opens the door for researchers to understand their samples from idiosyncratic to population levels, which enables a holistic perspective on the phenomena they’re investigating. The richness of qualitative data has always been appreciated but undervalued in many scientific arenas including psychology. Our approach takes one step toward providing researchers with a tool that can reclaim the utility of text data.

## Footnotes

1 We thank Denny Borsboom for bringing Guttman’s 1953 paper to our attention.
